# Supplementary material for: Direct coupling of light to valley current
Source: Nat Commun. 2024 Aug 31;15:7579. doi: 10.1038/s41467-024-51968-5 (PMC11365965; doi:10.1038/s41467-024-51968-5)
Supplement: Supplementary file 1 — Supplementary Information [file 41467_2024_51968_MOESM1_ESM.pdf]

Direct coupling of light to valley current: supplemental document

S. Sharma[1,2], D. Gill[1], J. Krishna[1] J. K. Dewhurst[3], and S. Shallcross[1]

[1]*Max-Born-Institut für Nichtlineare Optik und Kurzzeitspektroskopie, Max-Born-Strasse 2A, 12489 Berlin, Germany*

[2]*Institute for theoretical solid-state physics, Freie Universität Berlin, Arnimallee 14, 14195 Berlin, Germany*

[3]*Max-Planck-Institut fur Mikrostrukturphysik Weinberg 2, D-06120 Halle, Germany*

1 Intraband and total current: robustness of the ultrashort time current polarization and CEP control

Here we demonstrate that the key character of the ultrafast symmetry breaking regime of valley physics – the control of current via the orientability of the laser induced momentum trajectory, in turn controlled by pulse carrier envelope phase – is found both for the *intraband* current as well as in the full current, i.e. including both *inter-* and *intra*-band terms.

The current density generated by light can be broken down into intraband and interband contributions. We briefly review this fact. The macroscopic current density at time t can be written as

$$\mathbf{j}(t) = \frac{1}{V_{UC}} \sum_{\mathbf{q}} \langle \Psi_{\mathbf{q}}(t) | \boldsymbol{\nabla}_{\mathbf{q}} H(\mathbf{q}, t) | \Psi_{\mathbf{q}}(t) \rangle \tag{1}$$

where the sum $\mathbf{q}$ is over k-vectors in the Brillouin zone of area V_{UC}. Noting that the vector potential induces a evolution of crystal momentum given by the Bloch acceleration theorem:

$$\mathbf{k}(t) = \mathbf{q} - \mathbf{A}(t)/c, \tag{2}$$

with $\mathbf{q}$ the crystal momentum at $t = 0$, we may write the time dependent ket $|\Psi_{\mathbf{q}}(t)\rangle$ in the basis of instantaneous eigenvectors at $\mathbf{k}(t)$ as

$$|\Psi_{\mathbf{q}}(t)\rangle = \sum_i c_{i\mathbf{q}}(t) \left| \Phi_{i\mathbf{k}(t)} \right\rangle \tag{3}$$

where the instantaneous eigenvalues and eigenvectors are defined by

$$H(\mathbf{k}) \left| \Phi_{i\mathbf{k}} \right\rangle = E_i(\mathbf{k}) \left| \Phi_{i\mathbf{k}} \right\rangle \tag{4}$$

Taking the $\mathbf{q}$ derivative of the matrix element $\langle \Psi_{\mathbf{q}}(t) | H(\mathbf{q}, t) | \Psi_{\mathbf{q}}(t) \rangle$ leads directly to a separation into intra- and inter-band parts to the total current:

$$\mathbf{j}(t) = \mathbf{j}_{intra}(t) + \mathbf{j}_{inter}(t) \tag{5}$$

with

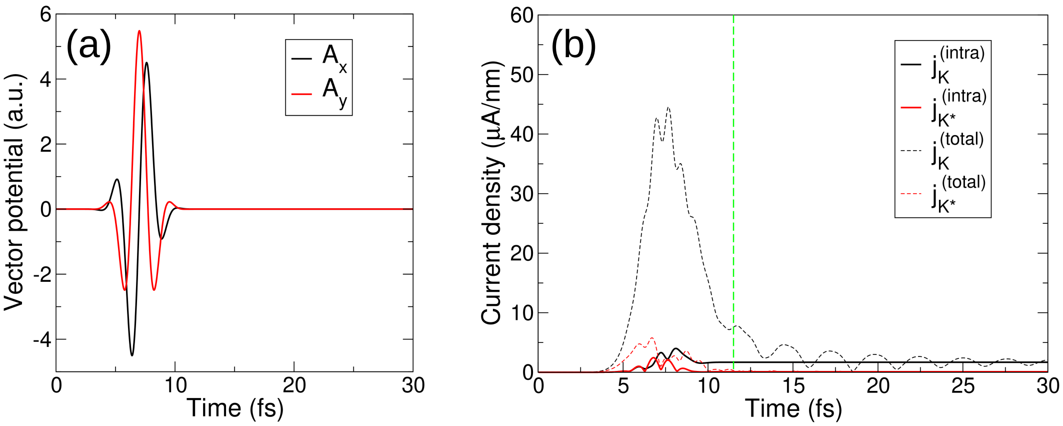

Fig. S1: The vector potential, $\mathbf{A}(t)$, of a few cycle circularly polarized light pulse is presented in panel (a), with the corresponding current shown in panel (b) where we display both the total current as well as its intraband component. Note that while these differ – the total exceeds the intraband – the same physics is seen: nearly complete valley polarization of the current generated on the same femtosecond time scale.

$$\mathbf{j}_{intra}(t) = \frac{1}{V_{UC}} \sum_{i\mathbf{q}} |c_{i\mathbf{q}}|^2 \, \boldsymbol{\nabla}_{\mathbf{k}} \, E_i(\mathbf{k})|_{\mathbf{k}=\mathbf{k}(t)} \tag{6}$$

and

$$\mathbf{j}_{inter}(t) = -\frac{1}{V_{UC}} \sum_{ij\mathbf{q}} c^*_{i\mathbf{k}(t)} c_{j\mathbf{k}(t)} E_j(\mathbf{k}(t)) \left\langle \boldsymbol{\nabla}_{\mathbf{p}} \Phi_{i\mathbf{k}(t)} \middle| \Phi_{j\mathbf{k}(t)} \right\rangle + c.c. \tag{7}$$

where we note that in both these expressions the sum over $\mathbf{q}$ enters through $\mathbf{k}(t)$, Eq. 2.

The intraband current depends on the occupations and Bloch velocities; the interband current, and hence the total, depends also on the phase structure of the time dependent wavefunction

acquired in the dynamical evolution induced by the light pulse. This latter contribution is oscillatory in character and decays with increasing time after the pulse as interference between many $\mathbf{k}$-vector dependent phases drives a reduction in overall amplitude. In experimental measurements at later times, therefore, it is the intraband current that plays the crucial role and this is the quantity presented in the manuscript. Here we show that all the basic physics of the intraband current manifest in the ultrashort time symmetry breaking regime also holds for the full current.

In Fig. 1 in panel (a) we present the vector potential of the lightform employed in the manuscript to explore the symmetry breaking regime for visible gap (transition metal dichalcogenide) systems. The valley integrated current magnitude is shown in panel (b), where one can note that although the total current is – due to transients from the interband contribution – larger than the intraband, the short time valley polarization found in the latter current is also found in the total. The total current oscillates with reducing intensity approaching the intraband current by ~ 30 fs.

A second crucial aspect of the light control explored in the manuscript is the direction control via the global carrier envelope phase (CEP) of the circularly polarized pulse that, in the short time limit, emerges as a relevant physical variable of the pulse. In Fig. 2 we show the dependence of the current components as a function of CEP, the pulse parameters as exactly those employed in Fig. 4 of the manuscript. Comparison of the same plot for the intraband component plotted in Fig. 4 of the manuscript reveals a similar CEP control in the total current, however the magnitude is increased and a phase shift can be noted such that the orientation of the current vector and the

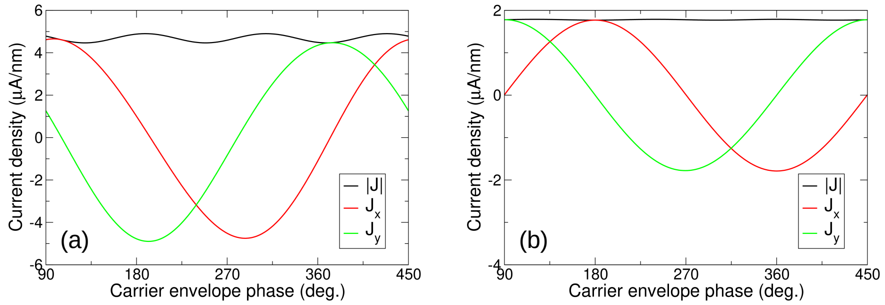

Fig. S2: *Carrier envelope phase control over the total current.* (a) The total current at 11.5 fs, indicated by the vertical line in Fig. 6, is shown as a function of carrier envelope phase with all other pulse parameters held fixed. Evidently complete control over total current direction can obtained via the carrier envelope phase. Except for a constant shift this is exactly the form found for the intraband component, panel (b).

underlying valley K-pole will now differ by a constant (but time dependent) angle.

2 The two band avoided crossing mode: dipole matrix elements

Here we consider the two band avoided crossing model, employed (alongside *ab-initio* theory) to study the transition metal dichalcogenides in the manuscript. In this section we will derive the dipole matrix elements, which play a crucial role in the observation of the short time symmetry breaking regime of circularly polarized light.

The Hamiltonian of the two band avoided crossing model (also known as gapped graphene) is given by

$$H(\mathbf{k}) = \begin{pmatrix} \Delta & t_{\mathbf{k}} \\ t^*_{\mathbf{k}} & -\Delta \end{pmatrix} \tag{8}$$

where Δ opens the gap and where if $t_{\mathbf{k}} = ke^{-i\nu\phi_k}$ then we have the gapped Dirac-Weyl Hamiltonian but we allow $t_{\mathbf{k}}$ to be more general (here the k-vector $\mathbf{k}$ has magnitude $k = |\mathbf{k}|$ and azimuthal angle $\phi_k = \tan^{-1} k_y/k_x$) and $\nu = \pm 1$ an integer that labels the two valleys. The eigenvalue problem presented by the Hamiltonian Eq. 8

$$H(\mathbf{k})a_\sigma = \varepsilon_\sigma(\mathbf{k})a_\sigma, \tag{9}$$

where $\sigma = \pm 1$ label the two eigenvectors and corresponding eigenvalue, can be diagonalized by the unitary transform

$$U = \frac{1}{\sqrt{2}} \begin{pmatrix} f_- & f_+ \\ -f_+e^{-i\theta} & f_-e^{-i\theta} \end{pmatrix} \tag{10}$$

where the columns are the eigenvectors

$$a_- = \frac{1}{\sqrt{2}} \begin{pmatrix} f_- \\ -f_+e^{-i\theta} \end{pmatrix} \quad \text{and} \quad a_+ = \frac{1}{\sqrt{2}} \begin{pmatrix} f_+ \\ f_-e^{-i\theta} \end{pmatrix} \tag{11}$$

and where we have defined the functions f_σ

$$f_\sigma = \sqrt{1 + \sigma \frac{\Delta}{\epsilon(\mathbf{k})}} \tag{12}$$

with

$$\epsilon(\mathbf{k}) = \sqrt{|t_\mathbf{k}|^2 + \Delta^2} \tag{13}$$

and the function $\theta(\mathbf{k})$

$$e^{i\theta(\mathbf{k})} = \frac{t_\mathbf{k}}{|t_\mathbf{k}|} \tag{14}$$

The corresponding eigenvalues are

$$\varepsilon_\sigma(\mathbf{k}) = \sigma\epsilon(\mathbf{k}) \tag{15}$$

and thus this model describes an avoided crossing of gap $E_{gap} = 2\Delta$ at $\mathbf{k} = \mathbf{0}$.

The function f_σ obeys the following relation under differentiation by $\mathbf{k}$:

$$\boldsymbol{\nabla}_\mathbf{k} f_\sigma = -\sigma \frac{\Delta}{2|t_\mathbf{k}|\epsilon} (\boldsymbol{\nabla}_\mathbf{k}\epsilon(\mathbf{k})) f_{-\sigma} \tag{16}$$

from which follows rules for differentiation of the eigenvectors

$$\boldsymbol{\nabla}_{\mathbf{k}} a_- = \frac{\Delta}{2\epsilon(\mathbf{k})|t_{\mathbf{k}}|}(\boldsymbol{\nabla}_{\mathbf{k}}\epsilon(\mathbf{k}))a_+ - i(\boldsymbol{\nabla}_{\mathbf{k}}\theta(\mathbf{k}))/\sqrt{2}\begin{pmatrix} 0 \\ \\ -f_+ e^{-i\theta(\mathbf{k})} \end{pmatrix} \tag{17}$$

and

$$\boldsymbol{\nabla}_{\mathbf{k}} a_+ = -\frac{\Delta}{2\epsilon(\mathbf{k})|t_{\mathbf{k}}|}(\boldsymbol{\nabla}_{\mathbf{k}}\epsilon(\mathbf{k}))a_- - i(\boldsymbol{\nabla}_{\mathbf{k}}\theta(\mathbf{k}))/\sqrt{2}\begin{pmatrix} 0 \\ \\ f_- e^{-i\theta(\mathbf{k})} \end{pmatrix} \tag{18}$$

Using these relations the dipole matix elements $d_{\sigma\sigma'}(\mathbf{k}) = a_\sigma^\dagger i \boldsymbol{\nabla}_{\mathbf{k}} a_{\sigma'}$ with $\sigma \neq \sigma'$ are found to be given by

$$\mathbf{d}_{-+}(\mathbf{k}) = -i\frac{\Delta(\boldsymbol{\nabla}_{\mathbf{k}}\epsilon(\mathbf{k}))}{2\epsilon(\mathbf{k})|t_{\mathbf{k}}|} - \frac{|t_{\mathbf{k}}|(\boldsymbol{\nabla}_{\mathbf{k}}\theta(\mathbf{k}))}{2\epsilon(\mathbf{k})} \tag{19}$$

and

$$\mathbf{d}_{+-}(\mathbf{k}) = i\frac{\Delta(\boldsymbol{\nabla}_{\mathbf{k}}\epsilon(\mathbf{k}))}{2\epsilon(\mathbf{k})|t_{\mathbf{k}}|} - \frac{|t_{\mathbf{k}}|(\boldsymbol{\nabla}_{\mathbf{k}}\theta(\mathbf{k}))}{2\epsilon(\mathbf{k})} \tag{20}$$

These are the dipole matrix elements presented in Fig. 3 of the manuscript in which are plotted the radio of the magnitudes $|\mathbf{d}_{-+}(\mathbf{k})|/|\mathbf{d}_{-+}(\mathbf{0})|$, with the magnitude defined by $\sqrt{(\mathrm{Re}\,\mathbf{d}_{-+})^2 + (\mathrm{Im}\,\mathbf{d}_{-+})^2}$ Similarly, the diagonal elements of the dipole matrix, $\mathcal{A}_\sigma(\mathbf{k}) = a_\sigma^\dagger i\boldsymbol{\nabla}_{\mathbf{k}} a_\sigma$ are given by

$$\mathcal{A}_\sigma(\mathbf{k}) = \frac{1}{2}(\boldsymbol{\nabla}_{\mathbf{k}}\theta(\mathbf{k}))\left(1 - \sigma\frac{\Delta}{\epsilon(\mathbf{k})}\right) \tag{21}$$

Employing the Dirac-Weyl approximation in which $t_{\mathbf{k}} = ke^{-i\nu\phi_k}$ we then have

$$\nabla_{\mathbf{k}}\epsilon = \frac{(\hbar v_F)^2 k}{\epsilon}\hat{\mathbf{k}} \tag{22}$$

$$\nabla_{\mathbf{k}}\theta = -\frac{\nu}{k}\hat{\boldsymbol{\phi}} \tag{23}$$

we then have for the dipole matrix elements

$$\mathbf{d}_{-+}(\mathbf{k}) = -i\frac{\hbar v_F \Delta}{2\epsilon^2}\hat{\mathbf{k}} + \frac{\nu\hbar v_F}{2\epsilon}\hat{\boldsymbol{\phi}} \tag{24}$$

with $\mathbf{d}_{+-}(\mathbf{k}) = \mathbf{d}_{-+}(\mathbf{k})^*$ and for Berry connection

$$\mathcal{A}_\sigma(\mathbf{k}) = -\frac{\nu}{2k}\left(1 - \sigma\frac{\Delta}{\epsilon}\right)\hat{\boldsymbol{\phi}} \tag{25}$$

and for the (out-of-plane) Berry curvature

$$\Omega_\sigma = -\frac{\sigma\nu(\hbar v_F)^2\Delta}{2\epsilon^3} \tag{26}$$

In a basis of Houston type states

$$|\phi_{n\mathbf{k}}\rangle = \exp\left(-\int^t dt'\,\varepsilon_{n\mathbf{k}}(t')\right)\exp\left(-\int^t dt'\,\mathbf{E}(t').\mathcal{A}(\mathbf{k}(t'))\right)e^{-i\mathbf{A}(t).\mathbf{r}}e^{i\mathbf{k}.\mathbf{r}}\,|u_{n\mathbf{k}}\rangle \tag{27}$$

with $\mathbf{A}(t)$ the time dependent vector potential and $|u_{n\mathbf{k}}\rangle$ the cell periodic function, then the time dependent Schrödinger equation can be written as

$$i\partial_t a = \mathbf{E}(t).\mathbf{d}\, a \tag{28}$$

where the matrix $\mathbf{d}$ contains only the off-diagonal dipole matrix elements $d_{nm} = (1-\delta_{nm})\,\langle\phi_{n\mathbf{k}}|\boldsymbol{\nabla}_{\mathbf{k}}|\phi_{m\mathbf{k}}\rangle$.

In the context of the two band model utilized in the manuscript this allows analysis of the dynamical trajectories of the symmetry breaking regime solely in terms of the interband dipole matrix element $\mathbf{d}_{-+}(\mathbf{k})$, see Fig. 3 of the manuscript.

3 Analysis of current for the two band avoided crossing model

In this section we provide an analysis of the origin of the time dependent phase shift in the carrier envelope phase dependence of the total current at early times for the few cycle symmetry breaking regime of circularly polarized light.

For the intraband current we always have $\theta_J = \phi_g$, where θ_J is the angle of the ultrafast light induced valley current and ϕ_g the emergent *global* carrier envelope phase of few cycle circularly polarized light. For the total current – including in the presence of excitonic effects – we find instead $\theta_J = \phi_g + \Delta\theta(t)$ with the shift $\Delta\theta(t)$ dependent on time.

To understand this we consider the minimal model of an avoided crossing described in the

previous section, and deploy the formulas for intra- and inter-band currents described in Sec. 1 of this supplemental.

We wish to consider the current that can be generated by an ultrafast laser pulse. To that end we consider the microscopic current of a state with crystal momentum $\mathbf{q}$ and at time t:

$$\mathbf{j}(\mathbf{q}, t) = \langle \Psi_{\mathbf{q}}(t) | \boldsymbol{\nabla}_{\mathbf{q}} H(\mathbf{q}) | \Psi_{\mathbf{q}}(t) \rangle \tag{29}$$

with the total macroscopic current density at time t obtained by integrating over the Brillouin zone:

$$\mathbf{j}(t) = \sum_{\mathbf{q}} j(\mathbf{q}, t) \tag{30}$$

As in Sec. 1 it is useful to transform the time dependent wavefunction to a basis of the instantaneous eigenkets given at the evolving crystal momentum

$$\mathbf{k}(t) = \mathbf{q} - \mathbf{A}(t)/c \tag{31}$$

For the avoided crossing model described in the previous section this is achieved via the unitary transform Eq. (10)

$$|\phi_{\mathbf{k}}(t)\rangle = U^{\dagger} |\Psi_{\mathbf{k}}(t)\rangle \tag{32}$$

Employing the chain rule in the manner described in Sec. 1 the intra-band ($\mathbf{j}_{intra}$) and inter-band ($\mathbf{j}_{inter}$) components of the total microscopic current $\mathbf{j}(\mathbf{q},t) = \mathbf{j}_{intra}(\mathbf{q},t) + \mathbf{j}_{inter}(\mathbf{q},t)$ are given by

$$\mathbf{j}_{intra}(\mathbf{q},t) = \sum_{\sigma} |c_{\mathbf{q}\sigma}(t)|^2 \boldsymbol{\nabla}_{\mathbf{k}}\, \varepsilon_{\sigma}(\mathbf{k})|_{\mathbf{k}=\mathbf{k}(t)} \tag{33}$$

where the $c_{\mathbf{q}\sigma}(t)$ represent the components of the time dependent wavefunction when expressed in the instantaneous eigenbasis:

$$|\phi_{\mathbf{q}}(t)\rangle = \begin{pmatrix} c_{\mathbf{q}-}(t) \\ c_{\mathbf{q}+}(t) \end{pmatrix} \tag{34}$$

The interband part is given by

$$\mathbf{j}_{inter}(\mathbf{k}) = -\langle\phi_{\mathbf{k}}(t)| \left((\nabla_{\mathbf{k}}U)^{\dagger}HU + U^{\dagger}H(\nabla_{\mathbf{k}}U)\right)|\phi_{\mathbf{k}}(t)\rangle \tag{35}$$

and using the unitary transform Eq. 10 we can write this in terms of the interband dipole matrix elements as

$$\mathbf{j}_{inter}(\mathbf{q},t) = \langle\phi_{\mathbf{q}}(t)|\, i2\epsilon(\mathbf{k}(t)) \begin{pmatrix} 0 & \mathbf{d}_{-+}(\mathbf{k}) \\ \mathbf{d}_{+-}(\mathbf{k}) & 0 \end{pmatrix} |\phi_{\mathbf{q}}(t)\rangle \tag{36}$$

where $\epsilon(\mathbf{k}(t))$ is defined in Eq. (13), and the dipole matrix elements $d_{\sigma\sigma'}$ in Eqs. (19) and (20). Note that the fact that $a_{\sigma}^{\dagger}\boldsymbol{\nabla}_{\mathbf{k}}a_{\sigma}$ is purely imaginary has been used to set the diagonal elements of this matrix to zero. Insertion of Eqs. (19) and (20) from Sec. 2 then yields a miscroscopic inter-band current composed of two distinct terms, out of phase by $\pi/2$:

$$\mathbf{j}_{inter}(\mathbf{q},t) = -\frac{\Delta}{|t_{\mathbf{k}(t)}|}(\boldsymbol{\nabla}_{\mathbf{k}}\epsilon(\mathbf{k}))|_{\mathbf{k}=\mathbf{k}(t)}\cos(2\epsilon(\mathbf{k}(t))t+\eta_{\mathbf{q}}(t)) + |t_{\mathbf{k}(t)}|(\nabla_{\mathbf{k}}\theta(\mathbf{k}))|_{\mathbf{k}=\mathbf{k}(t)}\sin(2\epsilon(\mathbf{k}(t))t+\eta_{\mathbf{q}}(t)) \quad (37)$$

where $2\epsilon(\mathbf{k}(t))$ is the local gap at $\mathbf{k}(t)$ (in atomic units this is thus the difference in dynamical phases) and $\eta_{\mathbf{k}}(t) = \arg(c_{\mathbf{q}+}(t)/c_{\mathbf{q}-}(t))$ a phase due to inter-band transitions; $t_{\mathbf{k}(t)}$, $\epsilon(\mathbf{k}(t)))$, and $\theta(\mathbf{k}(t))$ are defined in Eqs. (8), (13), and (14) respectively

The first of these terms is co-linear with the intra-band current, depending only on the gradient of the band manifold, while the second non-co-linear term depends on the gradient of the phase of the inter-sub-lattice hopping θ, $e^{i\theta(\mathbf{k})} = t_{\mathbf{k}}/|t_{\mathbf{k}}|$ see Eq. (8) of the previous section. In the Dirac-Weyl limit, close to the high symmetry K point, $\epsilon = v_F k$ and $e^{i\theta} = e^{-i\nu\phi_k}$ with $\phi_k = \tan^{-1} k_y/k_x$ the azimuthal angle of the crystal momentum. These two terms are thus orthogonal as, in polar coordinates, $\boldsymbol{\nabla}_{\mathbf{k}}\epsilon = (v_F, 0)$ and $\boldsymbol{\nabla}_{\mathbf{k}}\theta = (0, -\nu)$.

Thus at early times, when the intra-band and inter-band currents are both important, there will be two contributions to the “K-pole” induced valley current. One arises from intra- and inter-band terms in which the induced current at each $\mathbf{k}$-vector is proportional to $\mathbf{k}$, this is the term in which the current direction perfectly aligns with the carrier envelope phase angle i.e. is collinear with the K-pole moment, however there is a second term which (in the Dirac-Weyl limit) is exactly $\pi/2$ rotated from this first term. This will cause a rotation of the overall current vector from the CEP angle, which will be time dependent due to the time dependence of the magnitude of these contributions, as may be seen in Eq. 37.

4 Scaling current magnitude with pulse intensity

Pulse parameters explored in the phase diagrams presented in Fig. 5 of the paper were restricted to the duration and central frequency. Here we show that change of the pulse amplitude A_0 results only in a scaling of the current generated in the symmetry breaking regime, see Fig. 3 in which we present phase diagrams of current density magnitude and valley polarization for a range of laser pulse amplitudes. Note the different scales for the current density plots. Comparison of the phase diagrams for current density magnitude and its valley polarization with Fig. 5 confirms the robustness of the phase diagram structure to A_0.

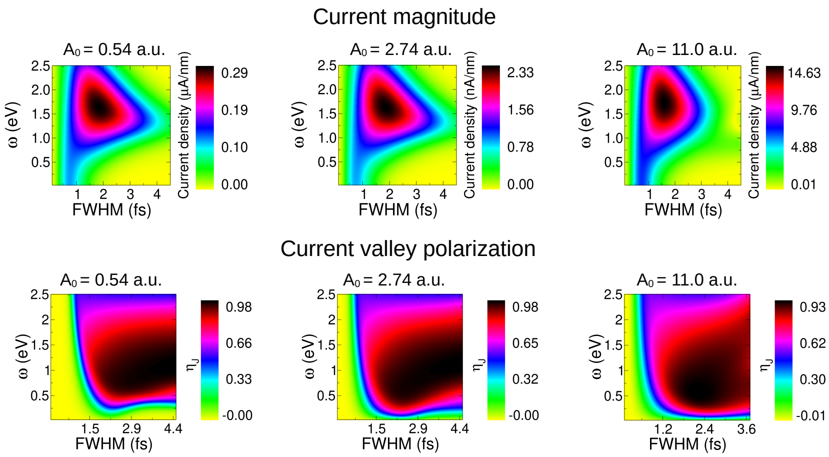

Fig. S3: *Pulse amplitude dependence of the symmetry breaking current created by circularly polarized light*. Shown is the current density magnitude and polarization as a function of pulse duration (full width half maxima, FWHM) and central frequency for three vector potential amplitudes A_0 = 0.54 a.u., 2.74 a.u., and 11.0 a.u. as labeled. Evidently the effect of the pulse amplitude is simply to scale the magnitude of the current created in the symmetry breaking regime of circularly polarized light.

5 Dependence of symmetry breaking current on pulse duration

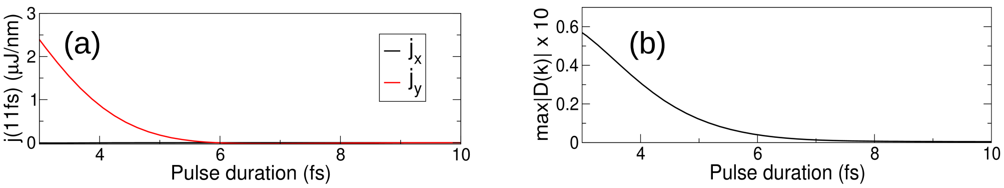

Fig. S4: Dependence of (a) the current and (b) the maximum amplitude of the $D(\mathbf{k})$ symmetry breaking density, for times that interpolate between the long time high symmetry regime and the short time symmetry breaking regimes shown in Fig. 1 of the manuscript. The current is seen to go continuously to zero, with $\max|D(\mathbf{k})|$ indicating that this symmetry breaking density is correlated to the short time emergence of a valley current generated by circularly polarized light.

In Fig. 1 of the manuscript we presented two limits of circularly polarized light: the many cycle long time limit (charge coupling only) and the few cycle limit (charge and current coupling). These describe, respectively, the high symmetry situation in which circularly polarized light can be described by a scalar, the helicity, and the short time symmetry breaking regime in which such pulses acquire vectorial character. Here we interpolate between these two limits, see Fig. 4.

In the manuscript the direction and magnitude of the short time emergent valley current created by few to single cycle circularly polarized light pulses was correlated with the symmetry breaking density, Eq. 1 of the manuscript and reproduced here:

$$D(\mathbf{k}) = |c_{\mathbf{k}}|^2 - \frac{1}{3}\sum_{i=1}^{3} |c_{M_i\mathbf{k}}|^2, \tag{38}$$

the sum is over three vectors related by the valley C_3 symmetry with M_i the 3 rotation operations of the C_3 group. This measures the deviation of the conduction band occupation at $\mathbf{k}$, $|c_{\mathbf{k}}|^2$, from the "star average" i.e the average over the vectors $\{M_i\mathbf{k}\}$, and thus measures the lowering of C_3 symmetry in the laser excited density. As can be seen in Figs. 4 the dependence of the amplitude of the symmetry breaking valley current follows very closely the maximum value of $|D(\mathbf{k})|$, indicating that this symmetry breaking density strongly correlates to the short time emergence of a valley current generated by circularly polarized light.

6 Dependence of symmetry breaking current on pulse amplitude

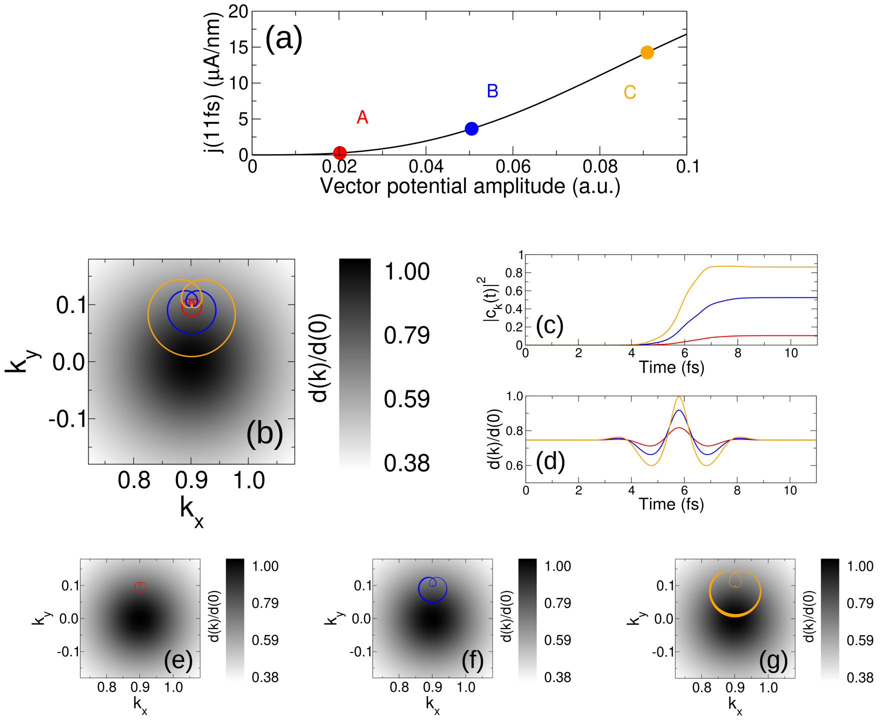

Fig. S5: (a) The symmetry breaking valley current induced by the single cycle circularly polarized pulse presented in Fig. 1(f-j) of the manuscript. The current is seen to increase with amplitude of the ultrafast pulse waveform, and for three representative values we plot in panel (b) a parametric plot of dynamical evolution of $\mathbf{k}(t)$ induced by the laser pulse for an initial k-vector $(0, +k_y)$. The corresponding time dependence of the conduction band occupation and the (normalized) magnitude of the dipole matrix element at $\mathbf{k}(t)$ are presented in panels (c) and (d) respectively, revealing that the greater the dipole matrix element "recorded" along the trajectory, $d(\mathbf{k})/d(\mathbf{0})$, the greater the conduction band occupation. Panels (e-g) display "fat trajectory" plots for each of the amplitudes A-C, in which the width of the trajectory lines indicates the rate of change of the occupation of the conduction band.

7 Time propagation of the von Neumann equation

Here we provide additional details of the time propagation of the von Neumann equation in the tight-binding method. In contrast to time-dependent density functional theory, in which all $\mathbf{k}$-vectors are coupled through the density, here each $\mathbf{k}$-vector is time propagated independently. The initial state is provided by a Fermi-Dirac distribution with $T = 0$, and following Stockman[1] we run the dynamics separately for each valance states.

The density matrix is evolved in the Bloch basis in which avoided crossing model and graphene Hamiltonians were presented in Methods (Eq. 5-7). For an initial $\mathbf{k}$-vector $\mathbf{k}(0)$ we diagonalize $H(\mathbf{k}(0))\,|\phi_i\rangle = \epsilon_i\,|\phi_i\rangle$ and for valence state i generate the density matrix $\rho(\mathbf{k}(0)) = |\phi_i\rangle\,\langle\phi_i|$. We then time propagate according to the von Neumann equation with each time step corresponding to value of the dynamically evolving crystal momenta $\mathbf{k}(t)$ according the Bloch acceleration theorem $\mathbf{k}(t) = \mathbf{k}(0) - \mathbf{A}(t)/c$.

$$\partial_t \rho(\mathbf{k}(t)) = -i\,[H(\mathbf{k}(t)), \rho(\mathbf{k}(t))] + \frac{1}{T_D}(\rho(\mathbf{k}(t)) - \mathrm{Diag}[\rho(\mathbf{k}(t))]) \tag{39}$$

To obtain the damping term $\frac{1}{T_D}(\rho(\mathbf{k}(t)) - \mathrm{Diag}[\rho(\mathbf{k}(t))])$ we at each time step transform $\rho(\mathbf{k}(t))$ from the Bloch basis to the local eigenbasis at $\mathbf{k}(t)$, subtract the diagonal elements, and transform back to the Bloch basis. Finally the initial condition loop $i = 1, n$ is summed over to obtain the final time dependent observables.

8 Time dependent density functional theory: treatment of excitonic physics

Here we present some further results of the calculations for WSe_2 in which excitonic physics was treated via the recently proposed Kohn-Sham-Proca scheme[2]. In Fig. 6 we present the laser pulse employed in our calculations for the case of zero carrier envelope phase (CEP), $\phi_g = 0$. The band structure and density of states for WSe_2 can be found in panels (a) and (b), respectively, of Fig. 7. The time-dependent density of states (TD-DOS) for the case $\phi_g = 0$ – other CEP show very similar results – is presented in Fig. 7c. Remarkably, we see that in the presence of excitons the TD-DOS is greater than in the absence of excitonic effects. This clearly indicates a strong field ionization of excitons, explaining the greater current densities found when the laser pumped dynamics include excitonic effects.

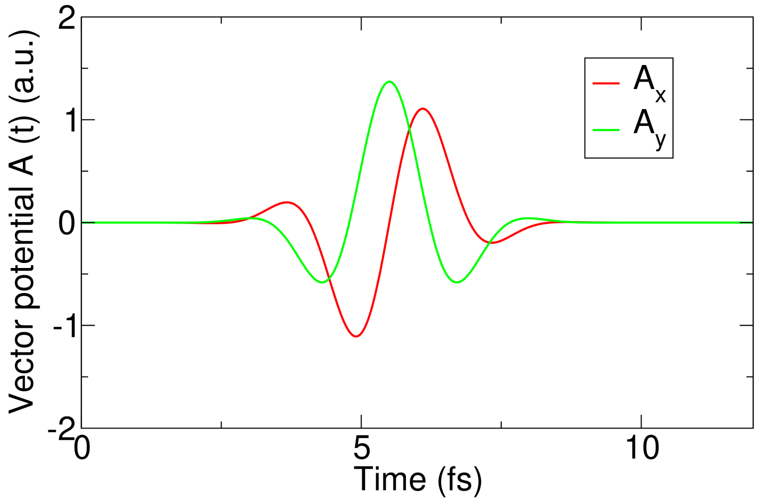

Fig. S6: The vector potential of the pulse used in the time-dependent density functional theory (TD-DFT) calculations.

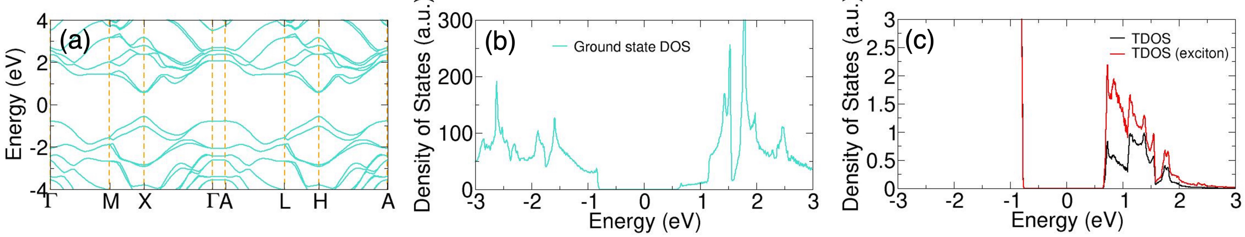

Fig. S7: The (a) band structure and (b) ground state density of states of WSe_2 as calculated in the local density approximation to density functional theory. (c) The time dependent excited state density of occupied states after excitation by the laser pulse displayed in Fig. 6, both with and without the inclusion of excitons in the dynamics.

1. Nematollahi, F., Apalkov, V. & Stockman, M. I. Phosphorene in ultrafast laser field. *Physical Review B* **97**, 035407 (2018). URL `https://link.aps.org/doi/10.1103/PhysRevB.97.035407`.

2. Dewhurst, J. K., Gill, D., Shallcross, S. & Sharma, S. Kohn-sham-proca equations for ultrafast exciton dynamics (2024). `2401.16140`.
